# Supplementary figures and images for: Chalkophore-mediated respiratory oxidase flexibility controls M. tuberculosis virulence
Source: eLife. 2025 Jun 5;14:RP105794. doi: 10.7554/eLife.105794 (PMC12140626; doi:10.7554/eLife.105794)

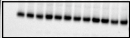

ALFA

Supplement: Figure 3—figure supplement 1—source data 1. [file elife-105794-fig3-figsupp1-data1.zip › 105794 Figure3-S1SourceData1/Fig 3-supp1 ALFA western uncropped label.pdf]

# RpoB

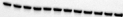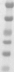

Supplement: Figure 3—figure supplement 1—source data 1. [file elife-105794-fig3-figsupp1-data1.zip › 105794 Figure3-S1SourceData1/Fig 3-supp1 rpoB western uncropped label.pdf]

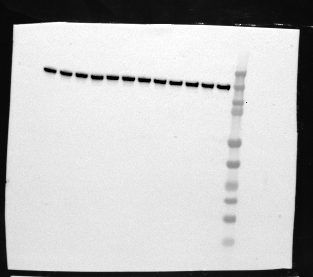

Supplement: Figure 3—figure supplement 1—source data 2. [file elife-105794-fig3-figsupp1-data2.zip › 105794 Figure3SourceData2/Fig 3-supp1 rpoB western uncropped.tif]

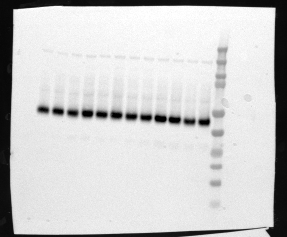

Supplement: Figure 3—figure supplement 1—source data 2. [file elife-105794-fig3-figsupp1-data2.zip › 105794 Figure3SourceData2/Fig 3-supp1 ALFA western uncropped.tif]
