# Supplementary material for: Chalkophore-mediated respiratory oxidase flexibility controls M. tuberculosis virulence
Source: eLife. 2025 Jun 5;14:RP105794. doi: 10.7554/eLife.105794 (PMC12140626; doi:10.7554/eLife.105794)
Supplement: Supplementary file 4. [file elife-105794-supp4.docx]

Supplemental file 4: Chemical Synthesis methods

Buglino et al

Supporting Information

A. Synthetic Methods S2

B. Synthesis of Diisonitrile **6** S3

C. ^1^H-NMR and ^13^C-NMR Spectra S7

# A. Materials and Methods

### Reagents

Reagents were obtained from Aldrich Chemical (www.sigma-aldrich.com) or Acros Organics (www.fishersci.com) and used without further purification. *N^2^,N^5^*-Bis(tert-butoxycarbonyl)-L-ornithine and L-phenylalaninol were obtained from AmBeed (www.ambeed.com) and used without further purification. Optima or HPLC grade solvents were obtained from Fisher Scientific (www.fishersci.com), degassed with Ar, and purified on a solvent drying system as described^[[1]](#footnote-1)^ unless otherwise indicated.

### Reactions

All reactions were performed in flame-dried glassware under positive Ar pressure with magnetic stirring unless otherwise noted. Liquid reagents and solutions were transferred through rubber septa via syringes flushed with Ar prior to use. Cold baths were generated as follows: 0 °C, wet ice/water.

### Chromatography

TLC was performed on 0.25 mm E. Merck silica gel 60 F254 plates and visualized under UV light (254 nm) or by staining with basic potassium permanganate (KMnO_4_), cerium ammonium molybdenate (CAM), or *p*-anisaldehyde. Silica flash chromatography was performed on E. Merck 230–400 mesh silica gel 60. Parallel chromatography was performed on an ISCO CombiFlash OptiX 10 instrument with RediSep silica gel normal phase columns with ELSD detection and UV detection at 254 nm.

### Analytical Instrumentation

NMR spectra were recorded on a Bruker UltraShield Plus 600 MHz Avance III NMR with DCH CryoProbe at 24 °C in CDCl_3_ unless otherwise indicated. Chemical shifts are expressed in ppm relative to TMS (^1^H, 0 ppm) or solvent signals: CDCl_3_ (^1^H, 7.26 ppm; ^13^C, 77.0 ppm), DMSO-*d_6_* (^1^H, 2.50 ppm; ^13^C, 39.52 ppm); coupling constants are expressed in Hz. NMR spectra were processed using Mnova (www.mestrelab.com/software/mnova-nmr). High resolution mass spectra were obtained on a Waters Acuity Premiere XE TOF LC-MS by electrospray ionization (ESI).

# B. Synthesis of Diisonitrile 6

Synthesis of diisonitrile 6. Boc = *t*-butyl­oxy­carbonyl; DIPEA = di­iso­propyl­ethyl­amine; DMAP = 4-di­methyl­amino­pyridine; NHS = *N*-hydroxy­succinimide; TFA = tri­fluoro­acetic acid; THF = tetra­hydro­furan.

***N^2^,N^5^*-Bis(*tert*-butoxycarbonyl)-l-ornithine *N*-hydroxysuccinimide ester (2, Boc-Orn[Boc]-NHS).** In a 50‑mL roundbottom flask, *N^2^,N^5^*-bis(*tert*-butoxycarbonyl)-l-ornithine (**1**) (2.0099 g, 6.04 mmol, 1.0 equiv) and dicyclohexylcarbodiimide (1.2932 g, 6.26 mmol, 1.03 equiv) were suspended in 20 mL anhyd THF and stirred at 0 °C for 10 min. *N*-Hydroxysuccinimide (699 mg, 6.07 mmol, 1.005 equiv) was added to the stirring solution in one portion. The mixture was stirred at 0 °C for 30 min, warmed to 22 °C, and stirred for 23 h. The mixture was filtered through a pad of celite and the solid white residue was washed with Et_2_O (40 mL). The combined filtrates were concentrated by rotary evaporation to afford the crude product as an off-white solid. Purification by silica flash chromatography (50% EtOAc in hexanes) yielded NHS ester **2** (2.1874 g, 5.09 mmol, 84%) as a white solid.

**TLC**: *R_f_* 0.43 (50% EtOAc in hexanes). **^1^H‑NMR** (600 MHz): δ 5.22 (d, *J* = 8.7 Hz, 1H), 4.77 (s, 1H), 4.64 (q, *J* = 7.2 Hz, 1H), 3.14 (q, *J* = 6.6 Hz, 2H), 2.81 (s, 4H), 1.99 – 1.90 (m, 1H), 1.86 – 1.78 (m, 1H), 1.63 (br s, 2H), 1.40 (m, 18H). **^13^C‑NMR** (150 MHz): δ 168.8, 168.4, 156.1, 155.0, 80.6, 79.2, 51.8, 39.9, 29.9, 28.5, 28.3, 25.6. **HRMS** (ESI) *m/z* calcd for C19H31N3O8Na ([M+Na]^+^) 452.2009; found 452.2000.

***N^2^,N^5^*-Bis(*tert*-butoxycarbonyl)-l-ornithyl-l-phenylalaninol (3, Boc-Orn[Boc]-Phin-OH).** In a 25‑mL roundbottom flask, l-phenylalaninol (148 mg, 0.978 mmol, 1.0 equiv) and DIPEA (405 μL, 2.94 mmol, 3.0 equiv) were dissolved in 10 mL anhyd THF and stirred at 0 °C for 10 min. *N^2^,N^5^*-Bis(*tert*-butoxycarbonyl)-l-ornithine *N*-hydroxysuccinimide ester (**2**) (495 mg, 1.15 mmol, 1.18 equiv) was added to the stirring solution in one portion. The mixture was stirred at 0 °C for 5 min, warmed to 22 °C and stirred for 17 h. The mixture was diluted with H_2_O and extracted with CH_2_Cl_2_ (3 x 20 mL). The combined organic extracts were washed with brine, dried (Na_2_SO_4_), filtered, and concentrated by rotary evaporation to afford the crude product. Purification by silica flash chromatography (50% EtOAc in hexanes) yielded the amide **3** (378 mg, 0.81 mmol, 83%) as a white solid.

**TLC**: *R_f_* 0.25 (50% EtOAc in hexanes). **^1^H‑NMR** (600 MHz): δ 7.30 – 7.25 (m, 2H), 7.24 – 7.19 (m, 3H), 6.59 (d, *J* = 8.2 Hz, 1H), 5.24 (d, *J* = 7.6 Hz, 1H), 4.80 – 4.74 (m, 1H), 4.20 – 4.08 (m, 2H), 3.66 (dd, *J* = 11.3, 3.5 Hz, 1H), 3.54 (dd, *J* = 11.5, 4.8 Hz, 1H), 3.21 (s, 2H), 3.01 (dt, *J* = 13.8, 6.7 Hz, 1H), 2.87 (td, *J* = 12.2, 7.5 Hz, 2H), 1.80 – 1.71 (m, 1H), 1.62 – 1.46 (m, 2H), 1.42 (s, 18H). **^13^C‑NMR** (150 MHz): δ 172.3, 156.6, 155.9, 137.9, 129.4, 128.7, 126.7, 80.2, 79.6, 63.6, 54.0, 53.1, 39.8, 37.1, 30.1, 28.6, 28.4, 26.2. **HRMS** (ESI) *m/z* calcd for C24H39N3O6Na ([M+Na]^+^) 488.2737; found 488.2749.

***N^2^,N^5^*-Bis(tert-butoxycarbonyl)-l-ornithyl-l-phenylalaninol acetate (4, Boc-Orn[Boc]-Phin-OAc).** In a 10‑mL roundbottom flask, *N^2^,N^5^*-bis(*tert*-butoxycarbonyl)-l-ornithyl-l-phenylalaninol (**3**) (324 mg, 0.696 mmol, 1.0 equiv), 4-(dimethylamino)pyridine (18 mg, 0.15 mmol, 0.21 equiv), and triethylamine (194 μL, 1.39 mmol, 2.0 equiv) were dissolved in 5 mL anhyd CH_2_Cl_2_. Acetic anhydride (91 μL, 0.962 mmol, 1.38 equiv) was added to the stirring solution and the mixture was stirred at 22 °C for 21 h. The reaction was quenched by addition of satd aq NaHCO_3_ and extracted with CH_2_Cl_2_ (3 x 20 mL). The combined organic extracts were washed with brine, dried (Na_2_SO_4_), filtered, and concentrated by rotary evaporation to afford the crude product. Purification by silica flash chromatography (50% EtOAc in hexanes) yielded the acetate **4** (325 mg, 0.64 mmol, 93%) as a white solid.

**TLC**: *R_f_* 0.50 (50% EtOAc in hexanes). **^1^H‑NMR** (600 MHz): δ 7.29 (t, *J* = 7.5 Hz, 2H), 7.24 – 7.17 (m, 3H), 6.50 (s, 1H), 5.08 (s, 1H), 4.67 (s, 1H), 4.40 (s, 1H), 4.12 (mf, 1H), 4.09 – 3.99 (m, 2H), 3.23 (s, 1H), 3.04 (dq, *J* = 12.5, 6.1 Hz, 1H), 2.84 (qd, *J* = 13.8, 7.1 Hz, 2H), 2.07 (s, 3H), 1.79 – 1.69 (m, 1H), 1.58 – 1.51 (m, 1H), 1.43 (m, 19H). **^13^C‑NMR** (150 MHz): δ 171.8, 171.3, 171.1, 156.5, 155.8, 137.1, 129.3, 128.8, 126.9, 80.1, 79.4, 64.8, 60.5, 53.6, 53.6, 49.7, 39.6, 37.6, 30.1, 28.6, 28.4, 26.4, 20.9, 14.3. **HRMS** (ESI) *m/z* calcd for C26H42N3O7 ([M+H]^+^) 508.3023; found 508.3023.

***N^2^,N^5^*-Bis((*R*)-3-isocyanobutanoyl)-l-ornithyl-l-phenylalaninol acetate (6, C4-Orn-Phin-OAc diisonitrile).** In a 20‑mL glass vial, *N^2^,N^5^*-bis(*tert*-butoxycarbonyl)-l-ornithyl-l-phenylalaninol acetate (**4)** (350 mg, 0.689 mmol, 1.0 equiv) was dissolved in 8 mL anhyd CH_2_Cl_2_ and stirred at 0 °C. Trifluoroacetic acid (2 mL, 25.6 mmol, 37.1 equiv) was added and the mixture was stirred at 0 °C for 5 min, warmed to 22 °C and stirred for 1 h. The mixture was concentrated by rotary evaporation to afford the crude bis(trifluoroacetate) salt (**5**).

In a 20-mL roundbottom flask, the crude bis(trifluoroacetate) salt (**5**) (369 mg, 0.689 mmol, 1.0 equiv) and DIPEA (1.08 mL, 6.20 mmol, 9 equiv) were dissolved in 5 mL anhyd CH_2_Cl_2_ and stirred at 0 °C. 2,5-Dioxopyrrolidin-1-yl (*R*)-3-isocyanobutanoate, prepared as previously described,^[[2]](#footnote-2)^ (367 mg, 1.75 mmol, 2.53 equiv) was added to the stirring solution and the mixture was stirred at 0 °C for 5 min, warmed to 22 °C and stirred for 17 h. The mixture was diluted with H_2_O and extracted with CH_2_Cl_2_ (3x20 mL). The combined organic extracts were washed with brine, dried (Na_2_SO_4_), filtered, and concentrated by rotary evaporation to afford the crude product. Purification by silica flash chromatography (100% EtOAc) yielded the diisonitrile **6** (212 mg, 0.43 mmol, 62%) as a colorless opaque solid.

**TLC**: *R_f_* 0.18 (100% EtOAc). **^1^H‑NMR** (600 MHz, DMSO-*d_6_*): δ 8.15 (d, *J* = 8.2 Hz, 1H), 8.02 (t, *J* = 5.7 Hz, 1H), 7.99 (d, *J* = 8.4 Hz, 1H), 7.27 (m, 2H), 7.20 (m, 3H), 4.25 (q, *J* = 7.6 Hz, 1H), 4.16 (q, *J* = 6.8 Hz, 1H), 4.06 (m, 2H), 4.01 (dd, *J* = 11.1, 4.7 Hz, 1H), 3.84 (dd, *J* = 11.1, 7.1 Hz, 1H), 3.03 (dh, *J* = 20.0, 6.7 Hz, 2H), 2.77 (dd, *J* = 13.9, 6.2 Hz, 1H), 2.72 (dd, *J* = 13.8, 7.8 Hz, 1H), 2.56 (dd, *J* = 14.6, 8.2 Hz, 1H), 2.49 – 2.35 (m, 3H), 1.99 (s, 3H), 1.56 (m, 1H), 1.50 – 1.33 (m, 1H), 1.30 (t, *J* = 7.1 Hz, 6H). **^13^C‑NMR** (150 MHz, DMSO-*d_6_*): δ 171.1, 170.3, 168.0, 167.9, 155.0, 154.9, 154.9, 137.9, 129.1, 128.2, 126.2, 64.6, 52.2, 48.9, 46.99, 46.95, 46.92, 42.2, 42.0, 38.2, 36.6, 29.9, 25.5, 20.99, 20.95, 20.6. **HRMS** (ESI) *m/z* calcd for C26H35N5O5Na ([M+Na]^+^) 520.2536; found 520.2536.

# C. ^1^H‑NMR and ^13^C‑NMR Spectra

1. Synthesis of Diisonitrile 6 S7

a. Boc-Orn(Boc)-NHS (**2**) S7

b. Boc-Orn(Boc)-Phin-OH (**3**) S8

c. Boc-Orn(Boc)-Phin-OAc (**4**) S9

d. C4-Orn-Phin-OAc Diisonitrile (**6**) S10

1. Pangborn, A. B.; Giardello, M. A.; Grubbs, R. H.; Rosen, R. K.; Timmers, F. J. *Organometallics* **1996,** *15*, 1518–1520. [↑](#footnote-ref-1)
2. Xu, Y.; Tan, D. S. *Org. Lett.* **2019**, *21*, 8731-8735. [↑](#footnote-ref-2)
